# A Chemoenzymatic Method To Systematically Quantify Core Fucosylation Stoichiometry of Glycoproteins and Reveal Its Roles in EMT and Embryonic Development

**DOI:** 10.1021/acs.analchem.5c05944

**Published:** 2026-01-19

**Authors:** Senhan Xu, Xing Xu, Kejun Yin, Ronghu Wu

**Affiliations:** School of Chemistry and Biochemistry and the Petit Institute for Bioengineering and Bioscience, 1372 Georgia Institute of Technology, Atlanta, Georgia 30332, United States

## Abstract

Core fucosylation of N-glycoproteins plays pivotal roles in regulating many cellular events such as receptor–ligand binding and cell adhesion. Here, we developed a chemoenzymatic method combining selective enrichment, enzymatic reactions, and multiplexed proteomics to systematically quantify the core fucosylation stoichiometries of glycoproteins in human cells. The results demonstrated that the core fucosylation stoichiometries vary dramatically in different subcellular compartments with the lowest in the lysosome and the highest in the extracellular matrix. Different core fucosylation stoichiometries were observed among glycosylation sites in various protein domains, and more aromatic and hydrophobic residues neighboring glycosylation sites are associated with lower core fucosylation stoichiometry. The method was applied to quantify the core fucosylation stoichiometry changes in the epithelial-to-mesenchymal transition (EMT), and some glycoproteins involved in extracellular matrix organization and ligand recognition displayed marked stoichiometry changes. Furthermore, the core fucosylation stoichiometries in embryonic human kidney cells (HEK293T) were compared with those in kidney cancer cells (A498). The average stoichiometry in HEK293T cells was much higher than that of A498 cells, indicating that core fucosylation may be a critical regulator in embryonic development. Without any sample restriction, this method can be extensively applied to investigate core fucosylation changes in various biological samples.

## Graphical abstract

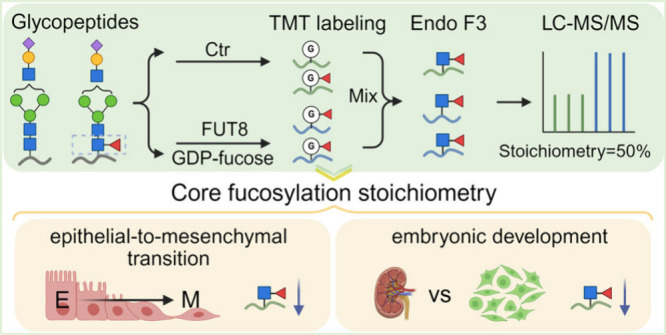

## Introduction

N-Glycosylation primarily occurs on proteins in the secretory pathway, regulating protein folding and trafficking, and different cellular events such as cell–cell communication and cell-matrix interactions.^1–6^ Aberrant N-glycosylation is directly related to human diseases, including cancer and neurodegenerative diseases.^7,8^ Fucosylation can occur on the innermost N-acetylglucosamine (GlcNAc) of N-glycan, which is catalyzed by a sole enzyme, α-(1,6)-fucosyltransferase (FUT8).^9^ The enzyme is localized in the Golgi apparatus and modifies complex and hybrid N-linked oligosaccharides post-translationally.^10^ Core fucosylation has been involved in various cellular processes, including ligand binding, immune recognition and response, cell adhesion, and the folding and stability of glycoproteins.^11,12^ The alteration of core fucosylation is implied in different diseases. For example, it is well-known that the presence or absence of core fucose on the Fc region of antibodies can affect their binding affinity to Fc receptors in immune cells, and altered fucosylation may impact antibody-dependent cellular cytotoxicity (ADCC) and other immune functions.^13^ Additionally, the expression of FUT8 is upregulated in many types of cancer, and core fucosylated glycoproteins can serve as biomarkers for detecting specific types of cancer.^7,14^ Moreover, it was observed that the level of core fucosylation on serum proteins was altered in chronic liver diseases.^15,16^

Given the importance of core fucosylation in regulating cellular activities and its implications in human diseases, it is imperative to monitor the level of core fucosylation to further understand its roles in physiological and pathological processes and to develop potential therapeutic strategies for disease treatments. Several methods were developed to address this need. For example, Cao et al. developed a sequential enzymatic method, in which enriched glycopeptides were sequentially treated with Endo F3 and PNGase F, to identify core fucosylation sites.^17^ In another study, a mutated Endo F3 (M-Endo-F3) that specifically recognizes truncated core fucosylated N-glycans by Endo F3 and catalyzes the addition of biotinylated oligosaccharides to the truncated glycosylation sites, enabling the detection of core fucosylation on the cell surface.^18^ The added tag can be subsequently cleaved using wild-type Endo F3, which leaves a relatively smaller mass tag for MS analysis. Furthermore, this mutated Endo F3 was also employed to globally analyze core fucosylation in HepG2, MCF7, and HeLa cells.^19^ One pivotal aspect for understanding the functions of core fucosylation is to measure its stoichiometry, i.e., the percentage of the same N-glycoprotein with core fucosylation. For example, a glycoprotein with the occupancy of core fucosylation of 10% or 90% in different conditions may indicate that it has distinct interactions with other molecules. Therefore, novel methods to accurately measure the core fucosylation stoichiometry will advance our understanding of this important modification. Despite that mass spectrometry (MS)-based proteomics has become very powerful to globally analyze protein modifications,^20–28^ and there are previous efforts on successful development of several proteomics methods to globally characterize core fucosylation,^17,29–32^ unbiased quantification of core fucosylation stoichiometry still remains quite challenging due to the glycan heterogeneity that may distort the quantification accuracy for different N-glycan types.

Here, we developed a novel chemoenzymatic method integrating selective enrichment, enzymatic reactions, and multiplexed proteomics to systematically quantify the core fucosylation stoichiometry of N-glycoproteins in human cells. First, we used dendrimer-conjugated benzoboroxole (DBA) to effectively capture glycopeptides. Second, the enriched glycopeptides were split into two equal halves. One half was treated with an excessive amount of FUT8 and GDP-fucose to modify all possible substrates of FUT8, while the other half was not added with FUT8. The accurate measurement of the abundance of core fucosylation in the treated half versus the untreated one allowed us to calculate the core fucosylation stoichiometry for a given glycosylation site. Third, the glycopeptides were further truncated with Endo F3, which is a highly specific endoglycosidase that cleaves within the chitobiose core of complex N-glycans to reduce the glycan size for improving the compatibility for MS analysis. Lastly, the control groups and the treated ones were labeled with the tandem mass tag (TMT) reagents, respectively, for MS quantification. We used this method to measure the core fucosylation stoichiometries in different samples to investigate the roles of core fucosylation in the epithelial-to-mesenchymal transition (EMT) and embryonic development. Without any sample restriction, this method can be extensively applied to study core fucosylation in different biological systems.

## Methods

### Cell Culture, Cell Lysis, and Protein Digestion

MCF10A, MCF7, MDA-MB-231, A498, HEK293T, and A549 cells (from the American Type Culture Collection (ATCC)) were grown in the media with the formulation suggested by ATCC. MCF10A cells were grown in MEGM Mammary Epithelial Cell Growth Medium (Lonza) supplemented with BPE, hEGF, insulin, hydrocortisone, cholera toxin, and 5% horse serum (Thermo). MCF7, MDA-MB-231, A498, A549, and HEK293T cells were grown in high glucose Dulbecco’s Modified Eagle’s Medium (DMEM, Sigma-Aldrich) containing 10% fetal bovine serum (FBS, Corning). All types of cells were cultured in a humidified incubator with 5.0% CO_2_ at 37 °C. Cells were harvested, washed twice with ice-cold phosphate-buffered saline (PBS), and pelleted by centrifugation at 500 g for 3 min. Cells were lysed in a buffer containing 50 mM HEPES, pH = 7.4, 150 mM NaCl, 0.5% sodium deoxycholate (SDC), 50 units/mL benzonase, and 1 tablet/10 mL EDTA-free protease inhibitor (Roche) for 45 min at 4 °C. The cell lysates were centrifuged for 10 min at 4696 g to remove the debris. Proteins were reduced by 5 mM dithiothreitol (DTT, Sigma) for 25 min at 56 °C and alkylated with 14 mM iodoacetamide (Sigma) for 20 min in the dark at room temperature. Proteins were purified by the methanol-chloroform protein precipitation method, and then proteins were digested with trypsin for 16 h at 37 °C.

### Treatment of A549 Cells To Induce EMT

A549 cells were passaged equally to 18 T175 flasks and grown until the density reached ∼50%. To acquire the mesenchymal A549 (A549 M) cells, A549 cells were treated with TGF-β for 120 h as reported previously,^33^ and the epithelial A549 (A549 E) cells were cultured for the same duration without treatment.

### Enrichment of Glycopeptides Using DBA

The enrichment of glycopeptides was achieved using the dendrimer-conjugated boronic acid derivative (DBA) method previously published from our lab.^34^ Digested peptides from each cell line, including epithelial and mesenchymal A549 cells, were dissolved in the binding buffer (DMSO, 0.5% TEA) and incubated for 30 min with the dendrimer-conjugated boronic acid-derivatized magnetic beads at room temperature. After incubation, the beads were washed with the washing buffer (50% H_2_O, 50% DMSO, 100 mM NH_4_OAc, pH = 10) four times, and enriched glycopeptides were eluted twice through the incubation with a solution containing ACN:H_2_O:TFA (50:49:1) at room temperature for 1 h. The enriched samples were lyophilized overnight for the subsequent enzymatic reactions.

### Optimizing Experimental Conditions for *in Vitro* Fucosylation

The amount of FUT8 used for the enzymatic reaction was optimized. To test the efficiency of fucosylation with different amounts of FUT8, triplicate experiments were performed for each amount (0, 2, 4, 8, 16, 25, 40 μg) of the enzyme added. Briefly, for each amount, each replicate contained around 100 μg glycopeptides. From each replicate, glycopeptides were dissolved in 100 mM HEPES, pH = 8.0. The solution was equally split to generate a sample with FUT8 (MedChemExpress) added and the other one as control without the addition of FUT8. Other than FUT8, all samples were added with 3 mM GDP-fucose and were diluted to the same volume. The samples were incubated at 37 °C overnight.

### *In Vitro* Fucosylation, TMT Labeling, and Deglycosylation by Endo F3

The enriched glycopeptides in triplicate experiments from different cell lines and conditions were dissolved in 100 mM HEPES, pH = 8.0, and were equally split with one-half for *in vitro* fucosylation and the other as control. In the samples for *in vitro* fucosylation, 16 μg FUT8 was added, and all samples were added with 3 mM GDP-fucose and diluted to the same volume. The reaction lasted overnight at 37 °C. Then the samples were desalted, and they were labeled using the TMT Sixplex reagents (Thermo), respectively. For each cell line and condition, three channels of TMT Sixplex were used to label peptides in the triplicate experiments for *in vitro* fucosylation samples, and the other three labeled the control samples. The labeling procedure was adapted from Zecha et al.^35^ with slight modifications. Briefly, the samples were dissolved in a buffer containing 33 μL 200 mM HEPES, pH = 8.6 and 10 μL ACN. The TMT reagents were dissolved in anhydrous ACN, and 100 μg TMT reagents were added to each sample. The reactions lasted for 1 h at room temperature, and they were quenched by adding 4 μL of 10% hydroxylamine (Sigma) for 15 min at room temperature. Then the samples from the same cell line or condition were combined, desalted, and lyophilized. The dried glycopeptides were resuspended in Glycobuffer 4, pH = 4.5 (NEB), and were deglycosylated using Endo F3 (NEB) for 3 h at 37 °C. The deglycosylated samples were dissolved in aqueous solution with 10 mM ammonium formate, pH = 10, and fractionated using C18-based reversed-phase chromatography (Agilent) with a Waters XBridge 3.5 μm C18 4.6 × 250 mm column. The mobile phase includes buffer A (10 mM ammonium formate, pH = 10) and buffer B (10% 10 mM ammonium formate, pH = 10, 90% ACN). The peptides were fractionated using a 45 min gradient. The fraction in every minute was collected over 10–50 min, and the fractions were combined into 12 samples. Each sample was cleaned up by stage-tip, lyophilized, and analyzed using LC-MS/MS.

### LC-MS/MS Analysis

Peptides were dissolved in a solution containing 5% ACN and 4% FA. For each sample, four microliters of the solution was loaded by a Dionex WPS-3000TPLRS autosampler (UltiMate 3000 thermostated Rapid Separation Pulled Loop Wellplate Sampler) onto a microcapillary column packed with C18 beads (Magic C18AQ, 1.9 μm, 200 Å, 75 μm x 16 cm, Michrom Bioresources). Peptides were separated using a nanoflow reversed-phase HPLC (Ultimate 3000 RSLCnano, Dionex). The buffer A was constituted with 0.125% formic acid, 2.5% ACN, and the buffer B contained 0.1% formic acid, 2.5% water in ACN. The peptides were eluted using a 95 min gradient at 0.3 μL/min. The microcapillary column was directly coupled for MS analysis using a Nanospray Flex ion source. The peptides were analyzed using an Orbitrap Exploris 480 mass spectrometer (Thermo). The full MS spectra were recorded using the following parameters: MS scan range: 350–1600 *m*/*z*; resolution: 120,000; Maximum injection time: Auto; normalized AGC: 100%. The precursor ions were selected for fragmentation by data-dependent acquisition (DDA). The most abundant precursor ions in each full MS scan were selected for fragmentation for 2 s at maximum. The following parameters were used for tandem MS scanning: Isolation width: 0.7 *m*/*z*; Isolation specificity: 90%; Resolution: 15,000; Normalized collision energy: 34%; AGC target: Standard; Max injection time: 25 ms. The selected ions were excluded for 60 s.

### Core Fucosylation Site Identification and Quantification

The raw files were converted into mzXML files, and then searched against the human (*Homo sapiens*) proteome database from UniProt using the SEQUEST algorithm (version 28).^36^ The following parameters were used during the search: 10 ppm precursor mass tolerance; 0.025 Da product ion mass tolerance; up to two missed cleavages; up to three modifications on each peptide; fixed modifications: TMT modification of lysine and the peptide N-terminus (+229.1629 Da). Variable modifications were set for the search of core fucosylated glycopeptides: Oxidation of methionine (+15.9949 Da); core fucosylated GlcNAc-linked asparagine after the truncation by Endo F3 (+349.1370 Da). False discovery rates (FDR) was controlled by a target-decoy method.^37^ Linear discriminant analysis (LDA) was employed to further improve the quality for peptide identification. The parameters included XCorr, ΔCorr, missed cleavages, adjusted PPM, fraction of ion matched, mass accuracy, peptide length, number of modifications per peptide, and charge states, and the peptide length should be more than seven amino acid residues. The FDR of glycopeptides was controlled to <1%.

The core fucosylation stoichiometry of each quantified glycopeptide was calculated by the relative intensities in the control groups divided by those in the treated groups. The median value was taken for the stoichiometry of all the same glycopeptides for every unique glycopeptide to calculate its stoichiometry.

### Data Analysis

The statistical analyses were performed using OriginLab. Protein clustering was performed using the DAVID platform (version 6.8).^38^ The information for protein secondary structures and disorder regions was predicted by an online server NetSurfP 3.0.^39^ The GRAVY score of protein and peptide sequences was calculated using the GRAVY calculator (https://www.gravy-calculator.de). The isoelectric points of proteins and peptides were calculated by IPC2.0.^40^ The sequence motifs surrounding the core fucosylated N-glycosylation sites were generated from pLogo.^41^ The information for protein domains was extracted from the SUPERFAMILY database.^42^ The figures for the workflow and subcellular organizations were generated by BioRender (https://www.biorender.com/).

## Results and Discussion

### Developing a Chemoenzymatic Method To Systematically Quantify the Core Fucosylation Stoichiometry

FUT8 is the only enzyme for catalyzing the core fucosylation of the innermost GlcNAc in complex N-glycans.^12^ To systematically quantify the core fucosylation stoichiometry, we developed a method coupling selective enrichment, enzymatic reactions, and multiplexed proteomics (Figure 1A). First, we applied an enrichment method developed in our lab that is based on synergistic and reversible covalent interactions between dendrimer boronic acid and glycans to effectively enrich glycopeptides.^34^ This method is highly effective, and it enabled us to identify 4195 N-glycosylation sites in mouse brain and 4691 N-glycosylation sites in human cells.^34^ Enriched glycopeptides were split into two equal halves. It was intended to measure the fraction of the copies of core fucosylation occurred physiologically. This was accomplished by treating one of the two halves with sufficient amount of FUT8 and GDP-fucose so that all possible substrates would be fucosylated. While in the other half without the FUT8 treatment, the glycopeptides were not further modified (Figure 1B). To achieve the complete core fucosylation of all possible substrates, we optimized the amount of FUT8 for the *in vitro* glycosylation (Figure S1). It was found that when the amount of FUT8 added reached 16 μg (in 200 μL reaction mixture), the ratio of *in vitro* core fucosylation was stable, demonstrating that the amount was sufficient to effectively catalyze the enzymatic reaction. With the optimal conditions, we performed the enzymatic reaction for *in vitro* core fucosylation in the treated group. Then both the treated and control groups were labeled using the TMT reagents, respectively.

**Figure 1:**
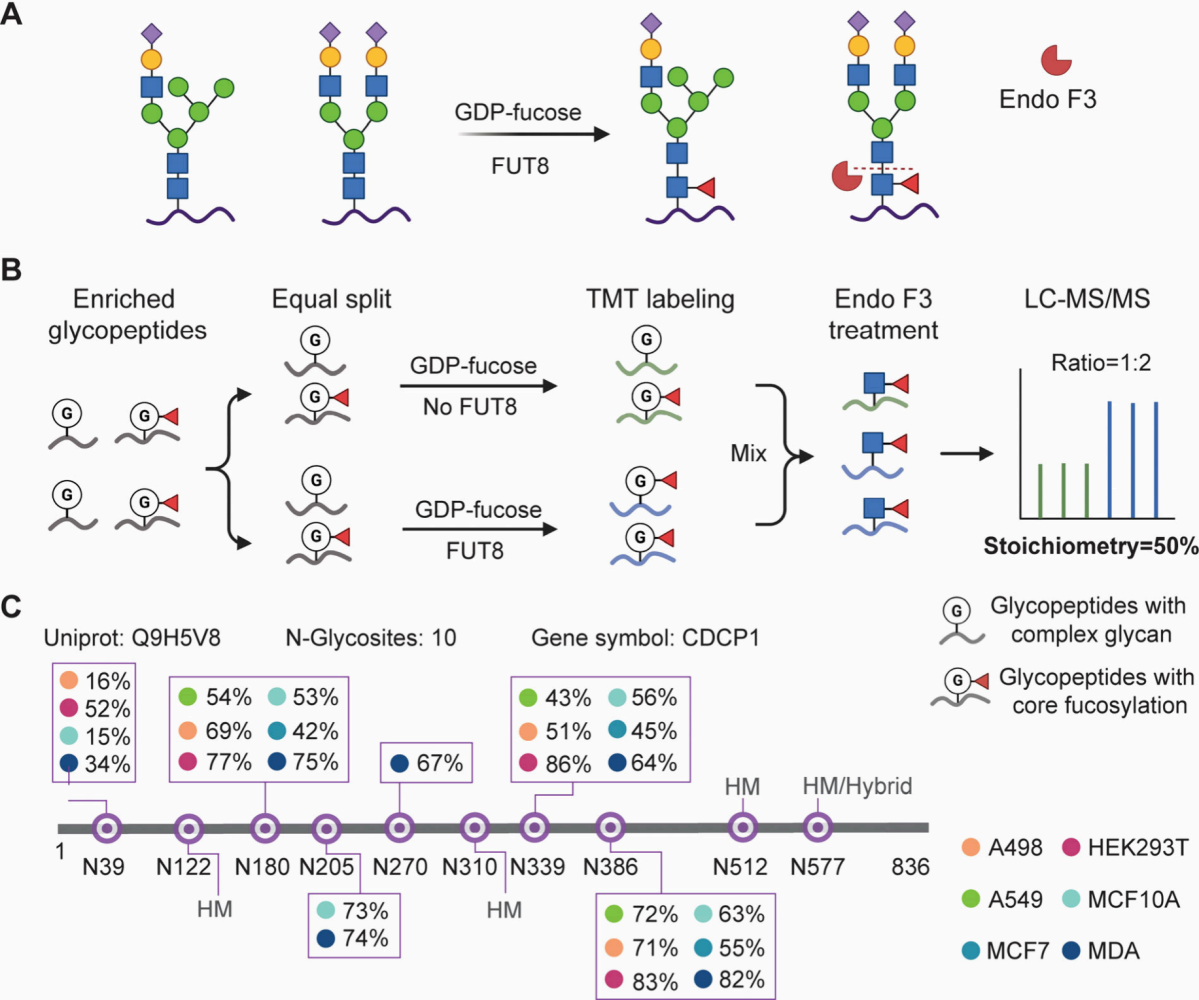
Experimental procedure for quantifying the core fucosylation stoichiometry in human cells and the stoichiometries measured in an example glycoprotein (CDCP1). (A) The enzymatic reaction to produce the core fucosylation of N-glycan by FUT8. (B) A workflow to measure the core fucosylation stoichiometry of N-glycosylation sites in human cells. (C) Core fucosylation stoichiometries of CDCP1 at six N-glycosylation sites across different cell lines. HM: high-mannose glycans.

The mixed glycopeptides from the two groups were treated with Endo F3, an endoglycosidase that cleaves fucosylated complex N-glycans, leaving a disaccharide tag containing a fucose and a GlcNAc that links to the asparagine residue of peptides (Figure 1A).^43^ This truncation simplifies the heterogeneity of N-glycans, benefiting the characterization and quantification of core fucosylation sites by MS.^17^ The core fucosylation stoichiometry was calculated based on the relative intensities of the TMT reporter ions (Figure 1B). Briefly, the copies of core fucosylated glycopeptides in the untreated group represented the number of actual core fucosylated glycopeptides, while the copies in the treated group represented the number of all possible core fucosylation events across the substrates of FUT8. The stoichiometry was determined by comparing the intensity of a glycopeptide in the untreated groups to the intensity of the same glycopeptide in the treated groups.

This method was applied to calculate the stoichiometries of glycopeptides in various cell lines and conditions, with the experiments being performed in biological triplicates. As an example, we quantified the core fucosylation stoichiometry of six N-glycosylation sites on CUB domain-containing protein 1 (CDCP1) across different cell lines (Figure 1C). CDCP1 contains ten identified N-glycosylation sites. Among them, 4 sites were not identified with core fucosylation, and they happen to be all annotated in the GlyGen database as high-mannose or hybrid-type glycans, which are not substrates of FUT8 and Endo F3.^44^ This observation strongly indicates the effectiveness of the identification of core fucosylation on complex N-glycans, and further demonstrates the applicability of our method for measuring the core fucosylation stoichiometries.

### Identification of Core Fucosylation Sites and Quantification of Their Stoichiometries in Human Cells

Using the optimized workflow, approximately 800 N-glycosylation sites with core fucosylation were identified in each cell line (Figure 2A), and the highest number of core fucosylation sites was observed in A549 cells. A total of ∼2000 core fucosylation sites were identified across different cell lines. These sites were assigned to more than 800 proteins, with approximately 500 proteins having core fucosylation sites in each cell line (Figure 2B). In each cell line, the stoichiometries were quantified in biological triplicates, and the average coefficient of variances (CVs) were lower or around 10%, showing reasonable reproducibility for the experiments (Figure S2). The average core fucosylation stoichiometry in various cell lines can be very different. In HEK293T and MDA-MB-231, the stoichiometry was over 80% on average, while in MCF7 the average stoichiometry was close to 50% (Figure 2C, Table S1).

**Figure 2:**
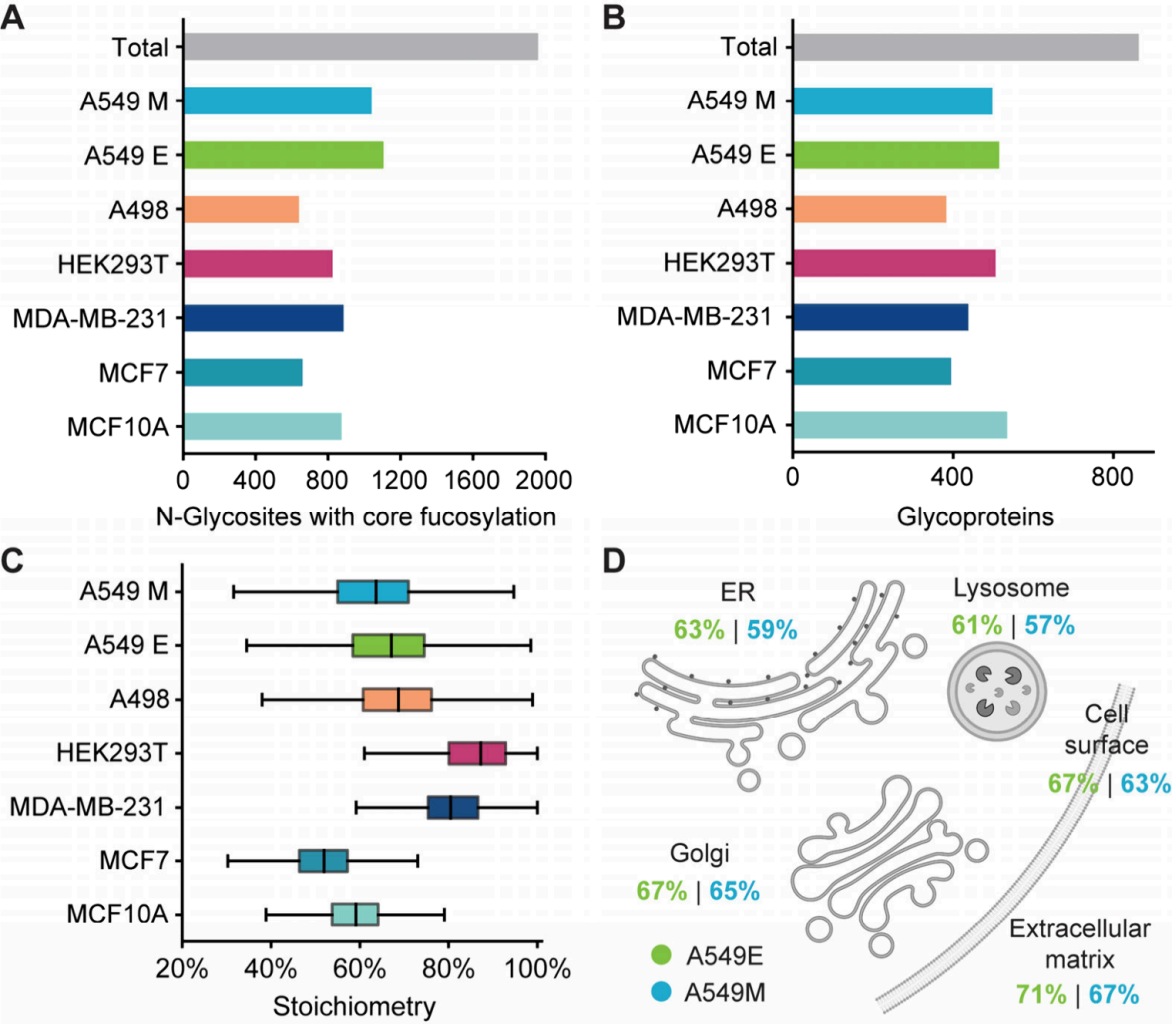
Identification of core fucosylation sites and quantification of their stoichiometries in human cells. (A–B) Identification of glycosites (A) and glycoproteins (B) with core fucosylation in human cell lines. (C) Box plot showing the core fucosylation stoichiometries quantified in different cell lines. (D) Average core fucosylation stoichiometry in different subcellular compartments in A549 cells. Box, 25th/75th percentiles; middle line, mean. Whiskers extend to highest/lowest values within 1.5 interquartile range (IQR).

Core fucosylation may be exclusive to a subset of N-glycoproteins, potentially favoring glycoproteins with specific localizations or functions. To test this, we compared proteins with core fucosylation to all N-glycoproteins identified in the same cell types. These data were derived from our previous work, which utilized DBA for glycopeptide enrichment prior to N-glycoprotein identification in HEK293T and MCF7 cells.^34^ The results showed that compared to all N-glycoproteins, glycoproteins with core fucosylation were highly associated with focal adhesion, plasma membrane, lysosome, and extracellular exosome (Figure S3). This finding indicates that core fucosylated glycoproteins are enriched in certain cellular compartments, which may be because that FUT8 is mainly localized to the Golgi apparatus,^45^ and glycoproteins in the downstream of secretory pathway will have a higher chance to be core fucosylated. Moreover, we observed that the core fucosylation stoichiometries for glycoproteins in various cellular compartments are quite different. In all cell lines studied, the average stoichiometry in the lysosome was the lowest, while the average stoichiometry for those in the extracellular matrix was the highest (Figure 2D and Figure S4). This indicates that the extent of core fucosylation can differ significantly depending on the cellular compartment.

### Site-Specific Regulation of Core Fucosylation Stoichiometries

To gain deeper insight into the characteristics of core fucosylation, we conducted the site-specific analysis. Initially, we compared the local structures of N-glycosylation sites with core fucosylation to the total N-glycosylation sites identified in MCF7 and HEK293T cells (Figure S5). The distribution of N-glycosylation sites, both with and without core fucosylation, was found to be similar across different secondary structures and disordered/ordered regions in both cell lines (Figure S5). This suggests that core fucosylation does not exhibit a strong preference for specific local structures of proteins. Moreover, the relative positions in full-length proteins were compared for N-glycosylation sites with core fucosylation and the total N-glycosylation sites (Figure S6). Our findings indicate that in both MCF7 and HEK293 cells, the distributions of relative positions are similar, implying that core fucosylation does not favor specific positions within a protein.

As core fucosylation occurs at the innermost GlcNAc of N-glycan, it is possible that the enzymatic activity is also affected by the local structure and the adjacent residues of the modified proteins. Our findings indicate that the core fucosylation stoichiometry was higher in the disordered regions than ordered ones (Figure 3A). When we compared the stoichiometries of core fucosylation on N-glycosylation sites across different local structures, including coil, sheet, and helix, we found the highest stoichiometry in coil (Figure 3B). This suggests that a higher stoichiometry of core fucosylation is associated with disordered regions, possibly because FUT8 can more easily access N-glycans with less steric hindrance. For example, we examined core fucosylation at six N-glycosylation sites on ITGA3 across multiple cell lines (Figure 3C). The site at N926, located within a β-sheet, consistently showed the lowest core fucosylation stoichiometry across different cell lines, further supporting the structural influence on enzymatic accessibility.

**Figure 3:**
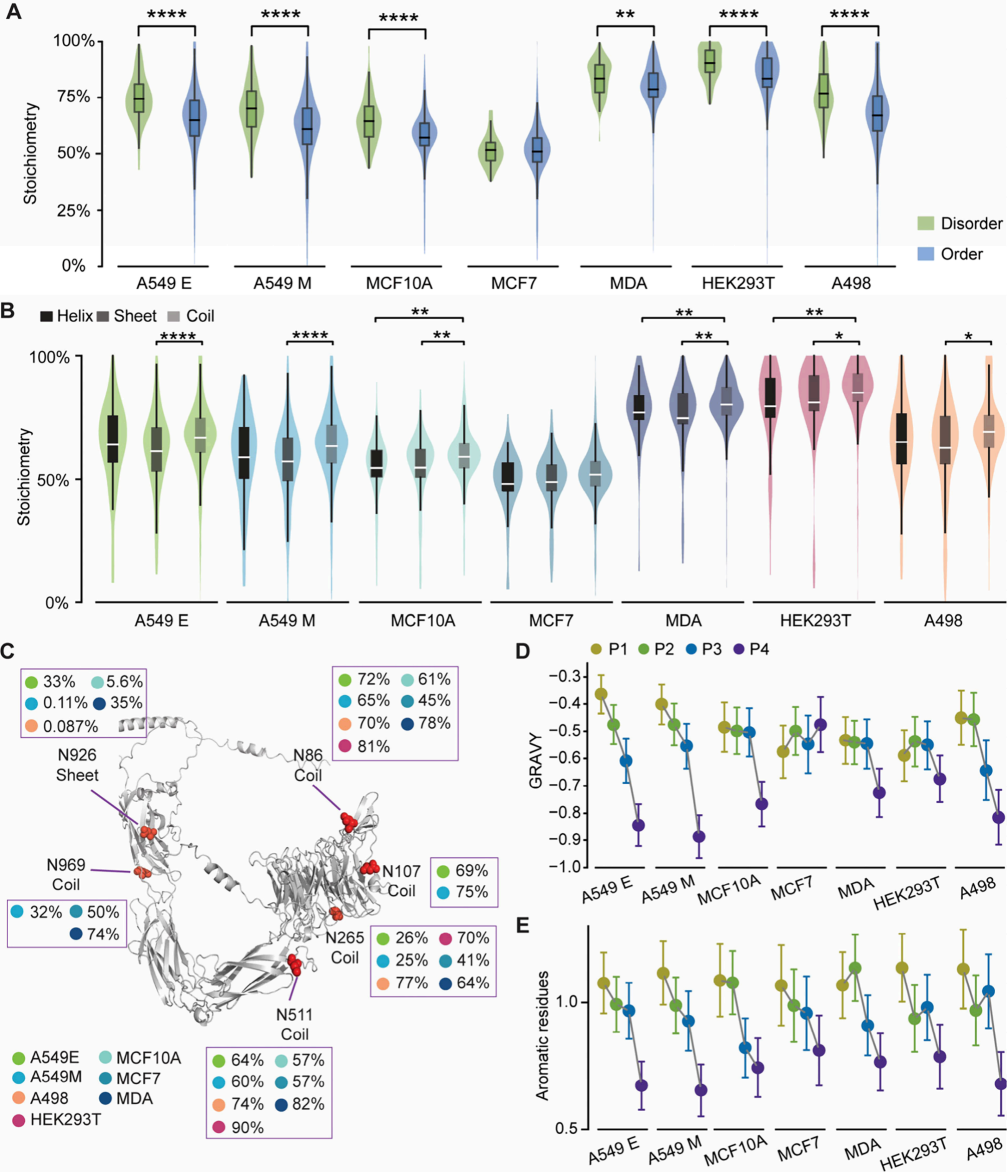
Site-specific analyses of core fucosylation stoichiometries in human cells. (A–B) Comparing the core fucosylation stoichiometries of N-glycosylation sites in disordered and ordered regions (A), and different secondary structures (B). (C) Predicted structure of ITGA3 from AlphaFold with core fucosylation stoichiometries mapped to individual N-glycosylation sites (highlighted as red spheres) across different cell lines. (D–E) Comparing the number of the GRAVY score (D) and aromatic residues (E) of the 13-mers constructed for the surrounding residues from P1–P4. Box, 25th/75th percentiles; middle line, mean. Whiskers extended to highest/lowest values within 1.5 interquartile range (IQR). The differences were assessed using Mann–Whitney U tests: *p < 0.05, **p < 0.01, ***p < 0.001, ****p < 0.0001.

Next, we observed significant variations in stoichiometries across different domains (Figure S7). For instance, the glycosylation sites in the growth factor receptor domain exhibited high core fucosylation stoichiometry. This aligns with previous reports that core fucosylation regulates epidermal growth factor receptor (EGFR)-mediated intracellular signaling by determining the binding of EGF to its receptors. Notably, when FUT8 was knocked out, EGFR phosphorylation was substantially blocked.^5^ We further explored the effect of surrounding amino acid residues on the stoichiometry of core fucosylation. N-glycosylation sites with core fucosylation were equally divided into four groups (P1 to P4), with P1 comprising those with the lowest stoichiometry and P4 having the highest. Interestingly, we observed an inverse relationship between the increase in stoichiometry and the decrease in hydrophobicity and aromaticity (Figure 3D-E).

To further elucidate which surrounding residues contributed most significantly to these changes, we constructed sequence motifs for the surrounding residues of N-glycosylation sites from P1 to P4 (Figure S8). We noted a general upregulation of aromatic residues in P1 across different cell lines, although this was not confined to a specific position. Similarly, the increased occurrence of charged residues in P4 was not limited to a particular position. Previous reports suggest that the likelihood of core fucosylation is enhanced when N-glycosylation occurs in a coil but is reduced with aromatic residues around,^46^ which aligns with our current findings. The association of charged residues with a higher stoichiometry of core fucosylation could be attributed to the fact that charged residues are more likely to be exposed to solvents, thereby increasing the accessibility of FUT8 to the glycosylation sites.^47^ Additionally, it was found that at the “+2” position, which is required to be S/T/C for N-glycosylation,^48^ T was overrepresented in P1. This could be due to the branched-out methyl group increasing the steric hindrance that prevents core fucosylation.

### Investigation of Core Fucosylation Stoichiometry Changes in EMT

EMT is a biological process that plays a crucial role in the development of various tissues and organs during embryogenesis and is also implicated in cancer progression and metastasis.^49^ During EMT, epithelial cells undergo molecular alterations to acquire a mesenchymal phenotype, which enables them to migrate and invade other tissues.^50^ A549 is a human lung adenocarcinoma cell line that is widely used for EMT studies.^51,52^ Under the treatment with TGF-β, the cell morphology undergoes significant changes over time and epithelial cells transitioned to mesenchymal cells (Figure S9).

Compared to the global N-glycoproteome of A549 cells, we found that approximately 43% of N-glycoproteins carry core fucosylation (Figure S10). These core fucosylated glycoproteins are predominantly localized to the Golgi apparatus, plasma membrane, and extracellular space (Figure 4A). In total, we quantified over 1,000 unique core fucosylated N-glycosylation sites in both the mesenchymal (M) and epithelial (E) states of A549 cells (Figure 4B). Importantly, when comparing core fucosylation stoichiometries with site-specific glycosylation abundance, we observed that the changes in core fucosylation stoichiometry are largely independent of the changes in glycosylation site abundance (Figure 4C). This finding highlights the unique regulatory role of core fucosylation that could be orthogonal to the other changes of N-glycosylation. The quantification results showed that the overall core fucosylation stoichiometries in both the epithelial and mesenchymal states were similar (66.8% vs 63.3%, Figure 4D and Table S1). Previous reports have indicated that FUT8 is upregulated in several cell lines following TGF-β treatment to induce EMT. Interestingly, our results demonstrate a slight decrease in the overall core fucosylation stoichiometry during EMT. However, the upregulation of FUT8 did not necessarily result in a higher amount of core fucosylation, which is also determined by the concentration of GDP-fucose and the correct localization of FUT8 for effective catalysis.^45^ Furthermore, the abundance of an enzyme may not be directly reflective of its activity.

**Figure 4:**
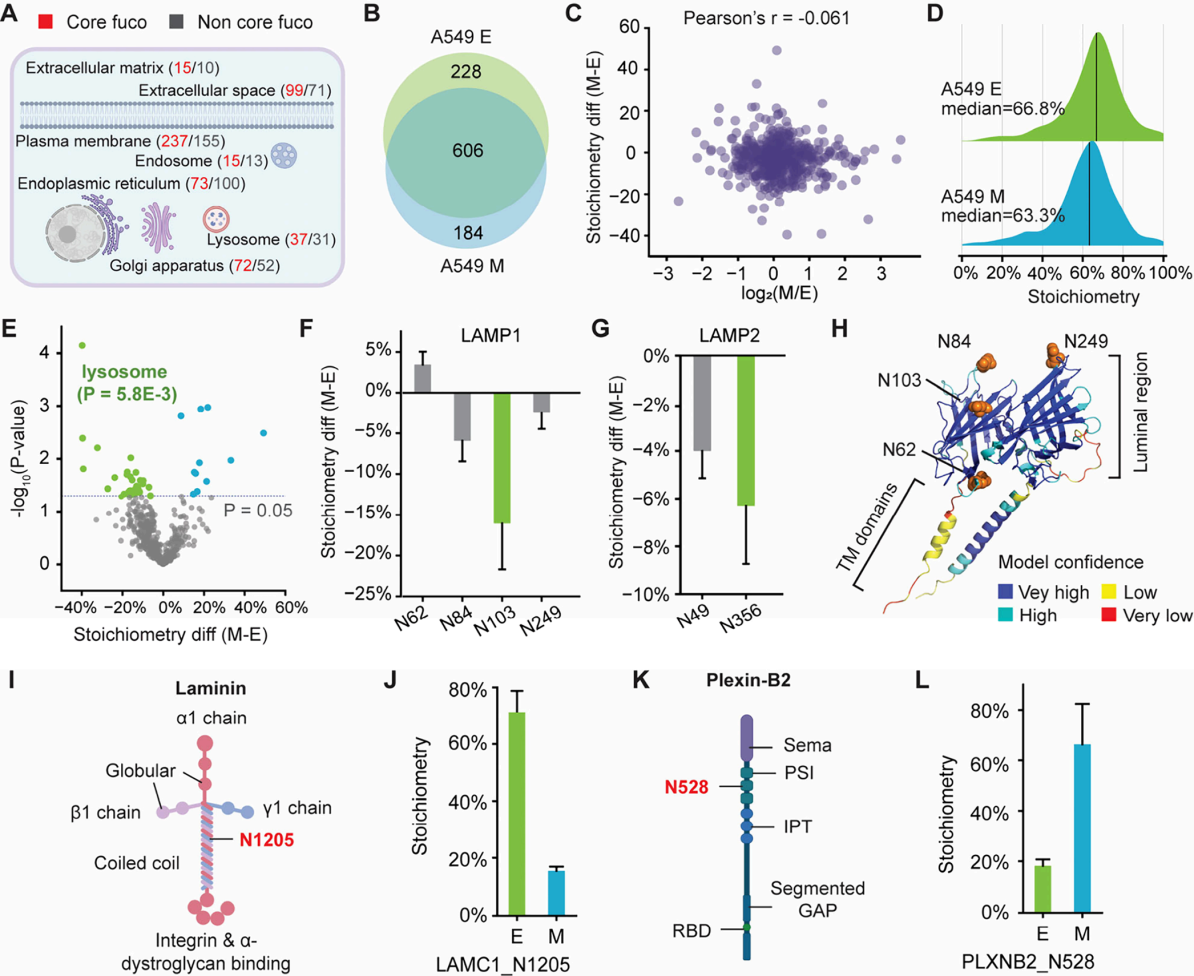
Investigation of core fucosylation stoichiometry changes in EMT. (A) Subcellular distributions of core fucosylated versus non-core fucosylated glycoproteins in A549 cells. (B) Overlap of unique core fucosylated N-glycosylation sites quantified in A549 epithelial (A549E) and mesenchymal cells (A549M). (C) Correlation between changes in N-glycosylation abundance and core fucosylation stoichiometry during EMT. (D) Comparison of core fucosylation stoichiometry distributions between A549 E and M cells. (E) Volcano plot illustrating site-specific differences in core fucosylation stoichiometry between A549 E and M cells. (F–G) Comparison of core fucosylation stoichiometry changes in LAMP1 (F) and LAMP2 (G) following EMT. (H) Glycosylated asparagine residues highlighted as orange spheres in the AlphaFold-predicted structure of LAMP1. (I) A cartoon shows the structure of laminin and the location of a glycosylation site (N1205) with core fucosylation. (J) Comparing the core fucosylation stoichiometries of N1205 on LAMC1 in A549 E and M cells. (K) The structure of plexin B2 and the location of a glycosylation site (N528) with core fucosylation. (L) Comparing the core fucosylation stoichiometries of N528 on plexin B2 in A549 E and M cells.

Furthermore, glycoproteins associated with functions such as response to hypoxia, proteolysis, and hydrolase activity exhibited relatively lower stoichiometry. In contrast, those related to cell differentiation, cell migration, and signal transduction demonstrated the highest stoichiometry (Figure S11). These observations could be attributed to the presence of numerous glycosidases in the lysosome that hydrolyze the glycan structure, including cleaving core fucosylation.^53^ Glycoproteins on the cell surface and in the extracellular matrix primarily facilitate ligand recognition and cell–cell interaction, strongly associated with the functions of core fucosylation.^11^ This could be the reason for higher core fucosylation stoichiometries on the cell surface and in the extracellular matrix. Interestingly, the pattern for the core fucosylation stoichiometries of glycoproteins with various functions differed between A549 E and A549 M cells. For proteins related to integrin binding, the core fucosylation stoichiometry in the mesenchymal state was much lower than that in the epithelial state. Conversely, for those related to cell-surface receptor signaling, the trend was reversed (Figure S11).

Stoichiometry changes of core fucosylation were systematically compared between the epithelial and mesenchymal states for all quantified N-glycosylation sites. Notably, several sites exhibited substantial differences in stoichiometry between the two states, with a marked decrease observed in glycoproteins associated with the lysosome following EMT (Figure 4D-E). For those with a significant decrease in stoichiometry when transitioning to the mesenchymal state, they were highly enriched in the lysosome and the phagosome. For example, LAMP1 and LAMP2—glycoproteins previously implicated in various types of cancer metastasis^54,55^ —showed significantly reduced core fucosylation stoichiometry at N103 and N356, respectively, in the mesenchymal state (Figure 4F-G). Interestingly, the decrease occurred despite an increase in the abundance of the parent proteins, particularly LAMP1, which exhibited more than a 1.5-fold increase during EMT. The quantified N-glycosylation sites are located in the luminal regions of LAMP1/2 (Figure 4H) and are not directly involved in the inhibition of the proton channel TMEM175.^56^ Therefore, the observed reduction in core fucosylation stoichiometry may instead influence the conformational stability or dynamics of luminal protein domains, potentially impacting lysosomal function during EMT.

Lastly, we examined the glycoproteins with the greatest stoichiometry change during EMT. For example, the stoichiometry of N1205 on laminin subunit gamma-1 (LAMC1) decreased almost 60% when transitioning to the mesenchymal state, while that of N528 on plexin-B2 (PLXNB2) increased by approximately 50% (Figure S12). Laminin is essential for the formation and function of basement membrane, and is composed of three subunits (α, β, and γ).^57^ Laminin plays vital roles in cell adhesion, migration, and differentiation.^58^ The short arms of laminins are composed of consecutive EGF domains that adopt a globular shape, and they bind to other laminin molecules (Figure 4I).^59^ The coiled region of the three laminin subunits intersects to form the long arm, which contains many disulfide bonds to help stabilize the interactions.^60^ The major function of the coiled region of laminin is to interact with the extracellular matrix and anchor laminin to the basement membrane.^61,62^ As N1205 of LAMC1 is localized in the coiled region, the core fucosylation of the N-glycan may affect the binding of laminin to other membrane molecules. When A549 cells were in the mesenchymal state, we found that the core fucosylation stoichiometry of N1205 on LAMC1 dropped from 70% in the epithelial state to only 10% (Figure 4J). It is possible that the removal of core fucosylation is related to the alteration of interactions between laminin and other surface molecules. This may contribute to the change of cell morphology to spindle-shaped that favors cell migration.^63,64^

Plexins are a family of transmembrane proteins involved in the regulation of cell adhesion, migration, and axon guidance.^65^ It acts as a receptor for the semaphorin family signal proteins, which is crucial for regulating cellular functions and cell–cell communication.^66^ The N-terminal region of plexin-B2 is extracellular, starting with a SEMA domain. This domain binds with semaphorins to mediate signal transduction. The SEMA domain is followed by the PSI domain, which binds with integrin. The IPT domain can bind transcription factors involved in gene transcription regulations (Figure 4K). We found that the core fucosylation stoichiometry of N528 on plexin B2 increased dramatically from approximately 20% to 60% during EMT (Figure 4L). The glycosylation site is localized in the PSI domain, suggesting that the upregulation of core fucosylation on the site may mediate the binding between plexin B2 and integrin. It was reported that plexin B2 orchestrated stem cell morphology and mobility by impacting the membrane association of β-catenin, focal adhesion, and integrin activation.^67^ The change in core fucosylation of N528 may affect the interaction between plexin B2 and integrin, impacting cell migration during EMT.

### The Stoichiometry of Core Fucosylation in HEK293T Cells Reveals Its Role in Embryonic Development

Core fucosylation was found to be involved in various aspects of embryonic development. For instance, the knockout of FUT8 in zebrafish embryos resulted in defective midline patterning during neuronal development.^68,69^ Additionally, the disruption of FUT8 induced severe growth retardation, early death during postnatal development, and emphysema-like changes in the lung.^70^ Therefore, a high level of core fucosylation may be essential for embryonic development. In this study, we quantified the core fucosylation stoichiometry of a human embryonic kidney cell line, HEK293T, and compared it with a cancerous cell line, A498, also isolated from kidney tissue. About 400 unique glycopeptides with core fucosylation were identified in both cell lines (Figure 5A, Table S1). The median stoichiometry of core fucosylation in HEK293T cells was 87%, which was much higher than that in A498 cells (Figure 5B), and is among the highest among all cell lines tested in this work (Figure 2C). As HEK293T is an embryonic cell line, this suggests that embryonic development is associated with higher core fucosylation stoichiometry.

**Figure 5:**
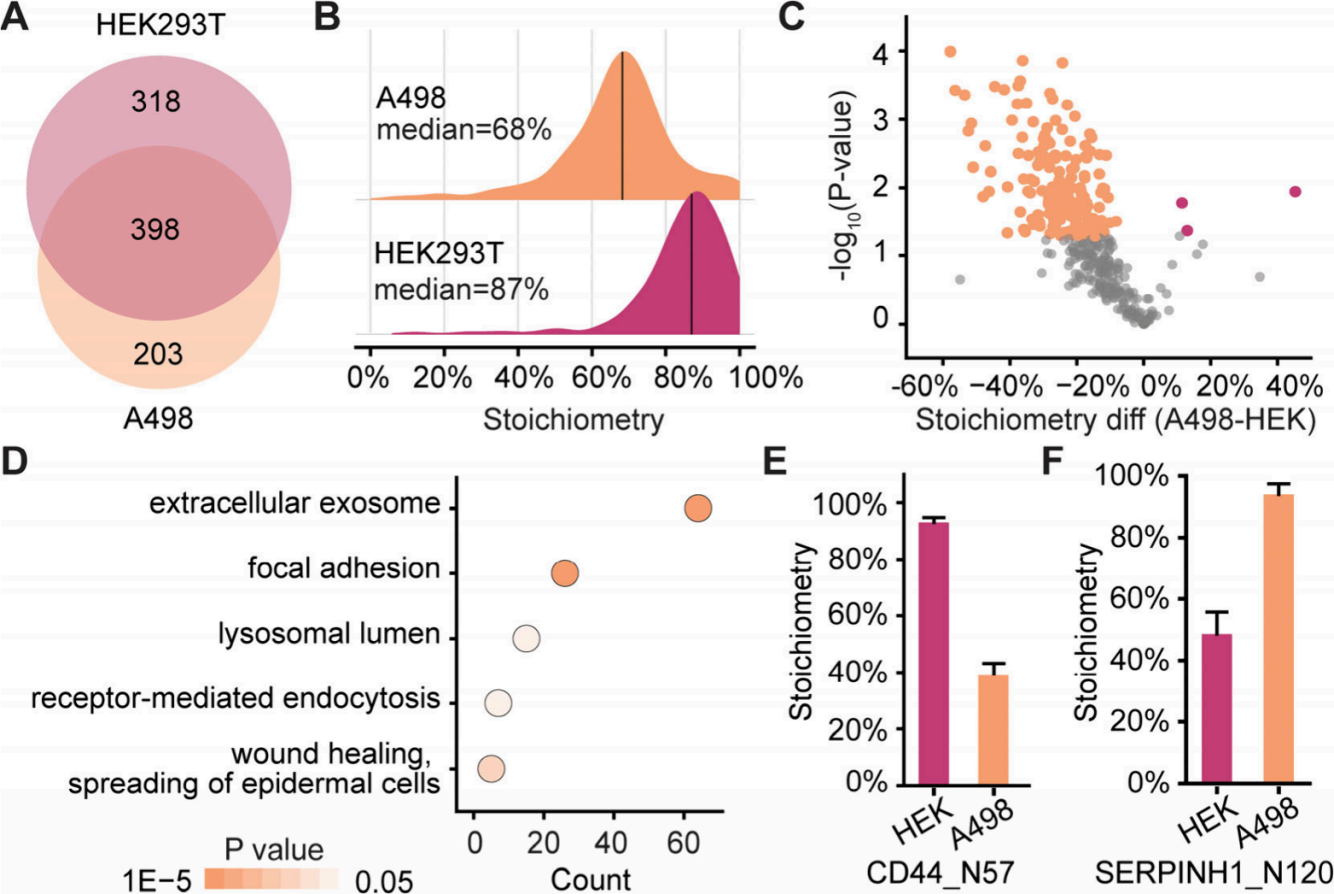
Investigation of core fucosylation stoichiometries in HEK293T and A498 cells. (A) The overlap of the unique glycopeptides with core fucosylation quantified in HEK293T and A498 cells. (B) Comparing the distributions of core fucosylation stoichiometries in HEK293T and A498 cells. (C) Volcano plot illustrating site-specific differences in core fucosylation stoichiometry between HEK293T and A498 cells. (D) Protein clustering shows functions of glycoproteins with decreased core fucosylation stoichiometries in A498 cell line. (E–F) The core fucosylation stoichiometries for the site N57 on CD44 (E) and the site N120 on serpin H1 (F) in HEK293T and A498 cells.

Notably, more than 40% of N-glycosylation sites exhibited a decrease in core fucosylation stoichiometry in A498 cells (Figure 5C). These glycoproteins are highly enriched in functions related to extracellular exosomes and focal adhesion (Figure 5D). For example, CD44 is a heavily glycosylated membrane receptor, and it is critical in cell adhesion, signal transduction, and cytoskeleton remodeling.^71^ It exhibited a marked increase in core fucosylation stoichiometry—from below 40% in A498 cells to over 90% in HEK293T cells (Figure 5E). It was reported that N-glycosylation can selectively block or enhance different receptor–ligand binding for CD44.^72^ These findings suggest that core fucosylation may directly regulate the binding affinity of CD44 to its ligands, thereby modulating signaling pathways involved in embryonic development. Moreover, the core fucosylation stoichiometry at N120 of serpin H1 was significantly higher in A498 compared to HEK293T cells (Figure 5F). Serpin H1 is an ER-resident chaperone essential for proper collagen folding.^73^ Given the central role of collagen in the extracellular matrix, increased core fucosylation stoichiometry of serpin H1 may influence collagen maturation, potentially affecting cell morphology and migration during embryonic development.^74^

## Conclusions

In this work, we developed a chemoenzymatic method for globally measuring core fucosylation stoichiometries. Using this method, we measured core fucosylation stoichiometries for N-glycosylation sites on over 1000 glycoproteins. It was found that the core fucosylation stoichiometry is associated with the subcellular localization, with glycoproteins in the lysosome having the lowest stoichiometries and those in the extracellular region having the highest. Furthermore, we found that glycosylation sites with more hydrophilic residues and fewer aromatic residues were more likely to have higher core fucosylation stoichiometry. This may be attributed to the structural preference of FUT8 in catalyzing core fucosylation.^46^ This method was then applied to investigate the changes of core fucosylation stoichiometries in EMT and embryonic development. Using TGF-β induced A549 cells as a model, we studied the stoichiometry changes in EMT. While the overall stoichiometry remained similar in the epithelial and mesenchymal states, certain proteins exhibited significant changes in core fucosylation stoichiometry during EMT. These proteins are known to play crucial roles in extracellular matrix organization and cell migration, suggesting that core fucosylation may be involved in extracellular matrix remodeling and cell migration regulation during EMT. Finally, we compared core fucosylation stoichiometries between an embryonic kidney cell line (HEK293T) and another kidney cell line (A498). The median core fucosylation stoichiometry was found to be much higher in HEK293T cells than A498 cells, suggesting that the increased core fucosylation stoichiometry in HEK293T cells may be associated with embryonic development. Our findings demonstrate that core fucosylation stoichiometry can vary significantly among different cell lines and may play key regulatory roles in various biological processes. The developed enzymatic method, free from sample restrictions, can be employed to measure core fucosylation stoichiometry in diverse biological systems, advancing our understanding of the biological roles of core fucosylation.

## Supplementary Material





## Data Availability

The raw files are available at https://massive.ucsd.edu/ProteoSAFe/dataset.jsp?accession=MSV000093936.

## Notes

Supplementary figures showing further results of core fucosylation level differences in various cellular compartments, protein domains, secondary structures and sequence motifs, and also other related analyses and results. Optimization of the amount of FUT8 for in vitro core fucosylation (Figure S1); comparison of the coefficient of variance (CV) for the core fucosylation stoichiometry in various cell lines (Figure S2); clustering for proteins with core fucosylation in HEK293T or MCF7 cells using the N-glycoproteome of HEK293T or MCF7 as the background (Figure S3); average core fucosylation stoichiometry in various cellular compartments (Figure S4); comparison of the distribution of N-glycosylation sites across local secondary structures (coil, sheet, helix) and structural order (ordered vs disordered regions) for all identified sites versus those exhibiting core fucosylation in MCF7 (A) and HEK293T (B) cells (Figure S5); comparison of the relative positions of all N-glycosylation sites versus core-fucosylated sites within their corresponding proteins in MCF7 (A) and HEK293T (B) cells (Figure S6); notched box plots showing the distribution of core-fucosylation stoichiometry for glycosylation sites within different protein domains in epithelial A549 cells (Figure S7); sequence motifs for core fucosylation sites with stoichiometry from low to high in various cell lines (Figure S8); A549 cell morphology change during EMT (Figure S9); overlap of glycoproteins with core fucosylation with the whole N-glycoproteome in A549 cells (Figure S10); protein clustering results show the stoichiometry for core fucosylation sites on proteins associated with different GO terms (Figure S11); stoichiometry changes for the N-glycosylation sites with core fucosylation quantified in both the epithelial and mesenchymal states (Figure S12) (PDF)
Quantification of core fucosylation stoichiometry of N-glycosylation sites in epithelial A549, mesenchymal A549, MCF10A, MCF7, MDA-MB-231, HEK293T, and A498 cells (Table S1) (XLSX)
