# Supplementary material for: A Chemoenzymatic Method To Systematically Quantify Core Fucosylation Stoichiometry of Glycoproteins and Reveal Its Roles in EMT and Embryonic Development
Source: Anal Chem. 2026 Jan 19;98(4):2968–79. doi: 10.1021/acs.analchem.5c05944 (PMC12874205; doi:10.1021/acs.analchem.5c05944)
Supplement: Supplementary file 1 [file ac5c05944_si_001.pdf]

Supporting Information

A Chemoenzymatic Method to Systematically Quantify Core Fucosylation Stoichiometry of Glycoproteins and Reveal Its Roles in EMT and Embryonic Development

Senhan Xu[#], Xing Xu[#], Kejun Yin, Ronghu Wu*

School of Chemistry and Biochemistry and the Petit Institute for Bioengineering and Bioscience, Georgia Institute of Technology, Atlanta, Georgia 30332, USA

# These authors contributed equally.

*Correspondence: ronghu.wu@chemistry.gatech.edu (R.W.)

Table of Contents

Fig. S1. Optimization of the amount of FUT8 for in vitro core fucosylation. S3

Fig. S2. Comparison of the coefficient variance (CV) for the core fucosylation stoichiometry in various cell lines. S4

Fig. S3. Clustering for proteins with core fucosylation in HEK293T or MCF7 cells using the N-glycoproteome of HEK293T or MCF7 as the background. S5

Fig. S4. Average core fucosylation stoichiometry in various cellular compartments. S6

Fig. S5. Comparison of the distribution of N-glycosylation sites across local secondary structures (coil, sheet, helix) and structural order (ordered vs. disordered regions) for all identified sites versus those exhibiting core fucosylation in MCF7 (A) and HEK293T (B) cells. S7

Fig. S6. Comparison of the relative positions of all N-glycosylation sites versus core-fucosylated sites within their corresponding proteins in MCF7 (A) and HEK293T (B) cells.. .... S8

Fig. S7. Notched box plots showing the distribution of core-fucosylation stoichiometry for glycosylation sites within different protein domains in epithelial A549 cells. S9

Fig. S8. Sequence motifs for core fucosylation sites with stoichiometry from low to high in various cell lines. S10

Fig. S9. A549 cell morphology change during EMT. S11

Fig. S10. Overlap of glycoproteins with core fucosylation with the whole N-glycoproteome in A549 cells. S12

Fig. S11. Protein clustering results show the stoichiometry for core fucosylation sites on proteins associated with different GO terms. S13

Fig. S12. Stoichiometry changes for the N-glycosylation sites with core fucosylation quantified in both the epithelial and mesenchymal states. S14

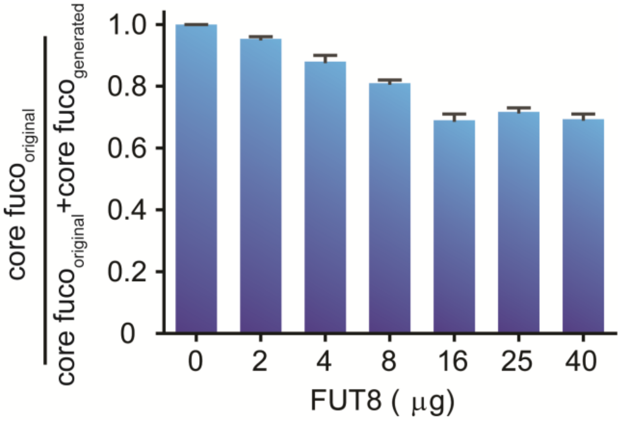

Fig. S1. Optimization of the amount of FUT8 for in vitro core fucosylation. core fuco$_{original}$: Relative abundance of core fucosylated peptides in the control group. core fuco$_{original}$+core fuco$_{generated}$: Relative abundance of core fucosylated peptides in the FUT8-treated group.

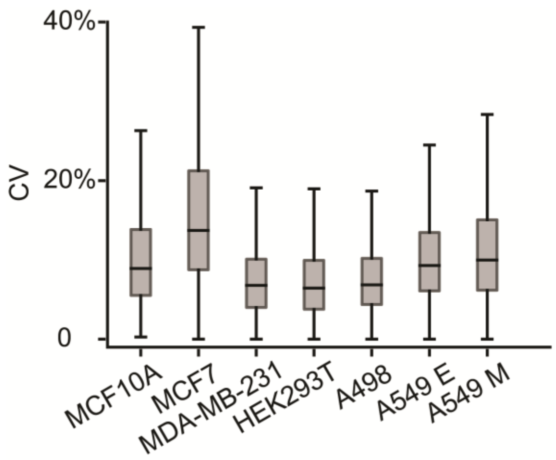

Fig. S2. Comparison of the coefficient variance (CV) for the core fucosylation stoichiometry in various cell lines. The CV values were calculated based on signal-to-noise (S/N) intensities. Interquartile ranges (IQRs) are shown as boxes with the median as a middle line and the whiskers extending up to 1.5-fold IQR.

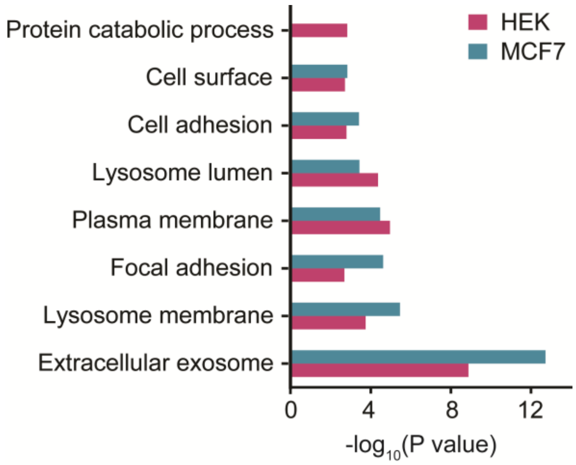

Fig. S3. Clustering for proteins with core fucosylation in HEK293T or MCF7 cells using the N-glycoproteome of HEK293T or MCF7 as the background.

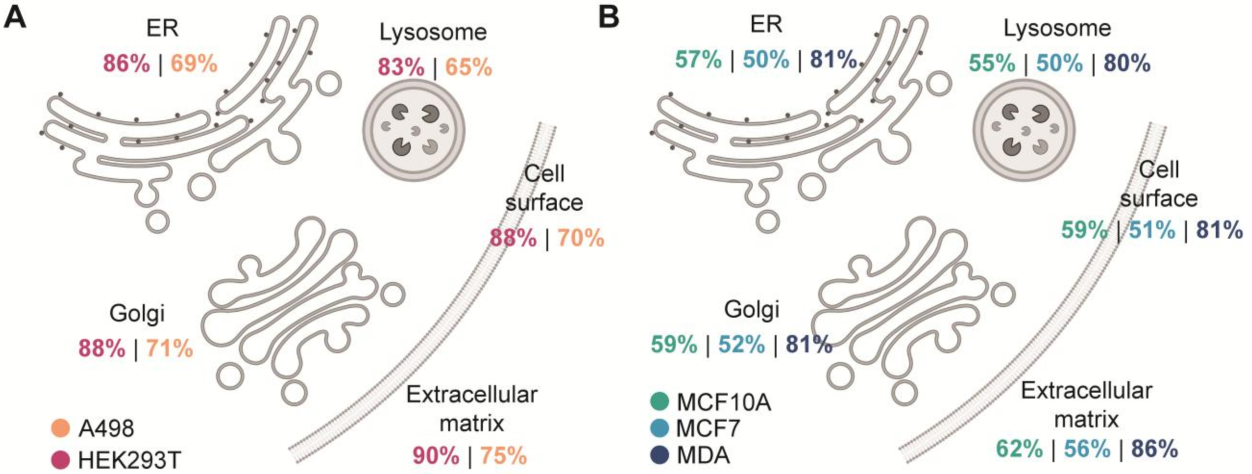

Fig. S4. Average core fucosylation stoichiometry in various cellular compartments. In HEK293T and A498 cells (A); in MCF10A, MCF7, and MDA-MB-231 cells (B).

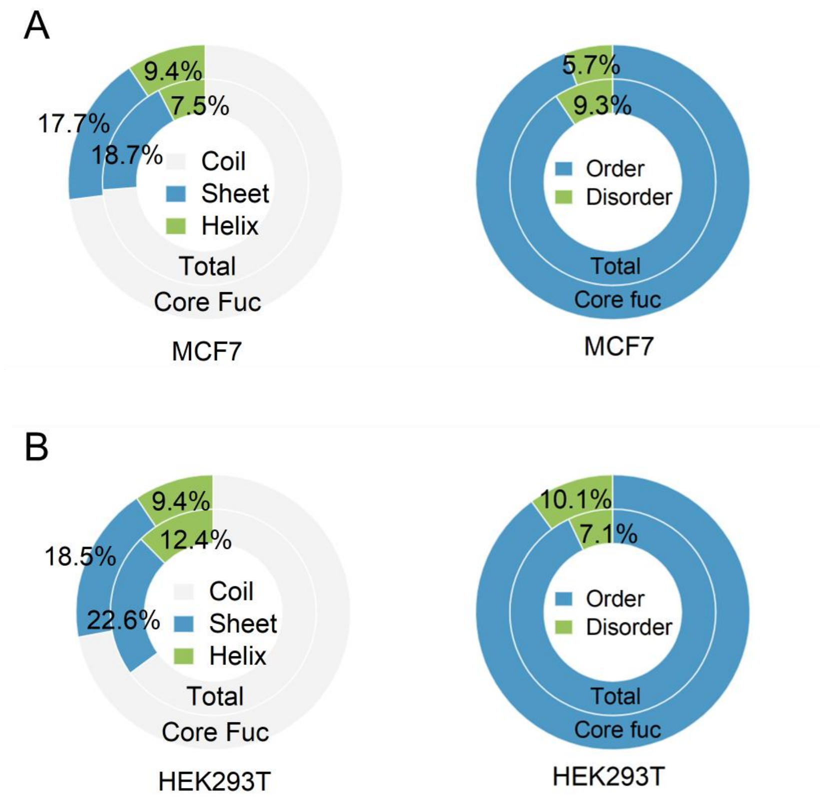

Fig. S5. Comparison of the distribution of N-glycosylation sites across local secondary structures (coil, sheet, and helix) and ordered vs. disordered regions for all identified sites versus those exhibiting core fucosylation in MCF7 (A) and HEK293T (B) cells.

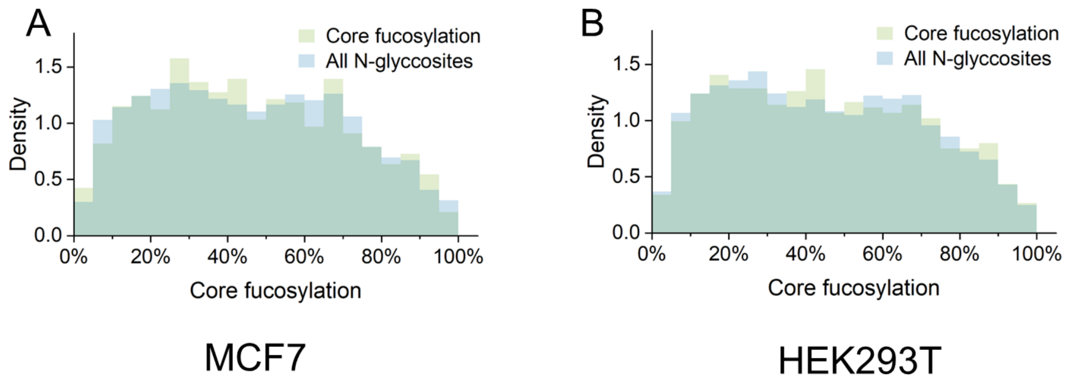

Fig. S6. Comparison of the relative positions of all N-glycosylation sites versus core-fucosylated sites within their corresponding proteins in MCF7 (A) and HEK293T (B) cells. The X-axis represents normalized protein length, where 0% denotes the N-terminus and 100% denotes the C-terminus.

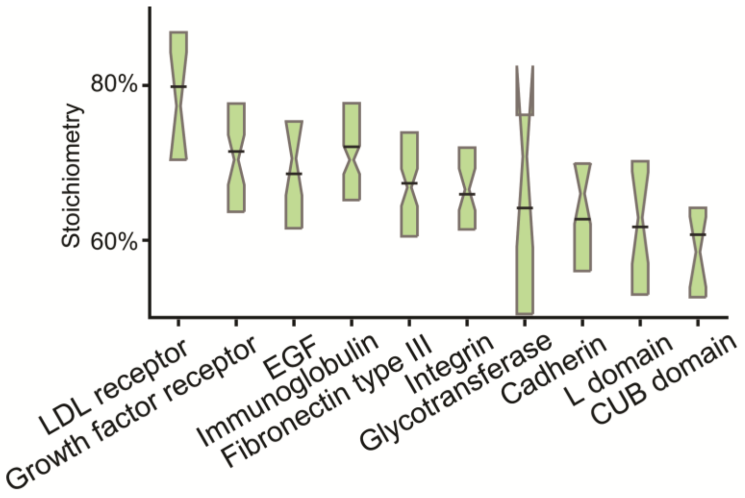

Fig. S7. Notched box plots showing the distribution of core-fucosylation stoichiometry for glycosylation sites within different protein domains in epithelial A549 cells. Box, 25th/75th percentiles; middle line, mean; the surrounding violin shape represents the density distribution of stoichiometry values.

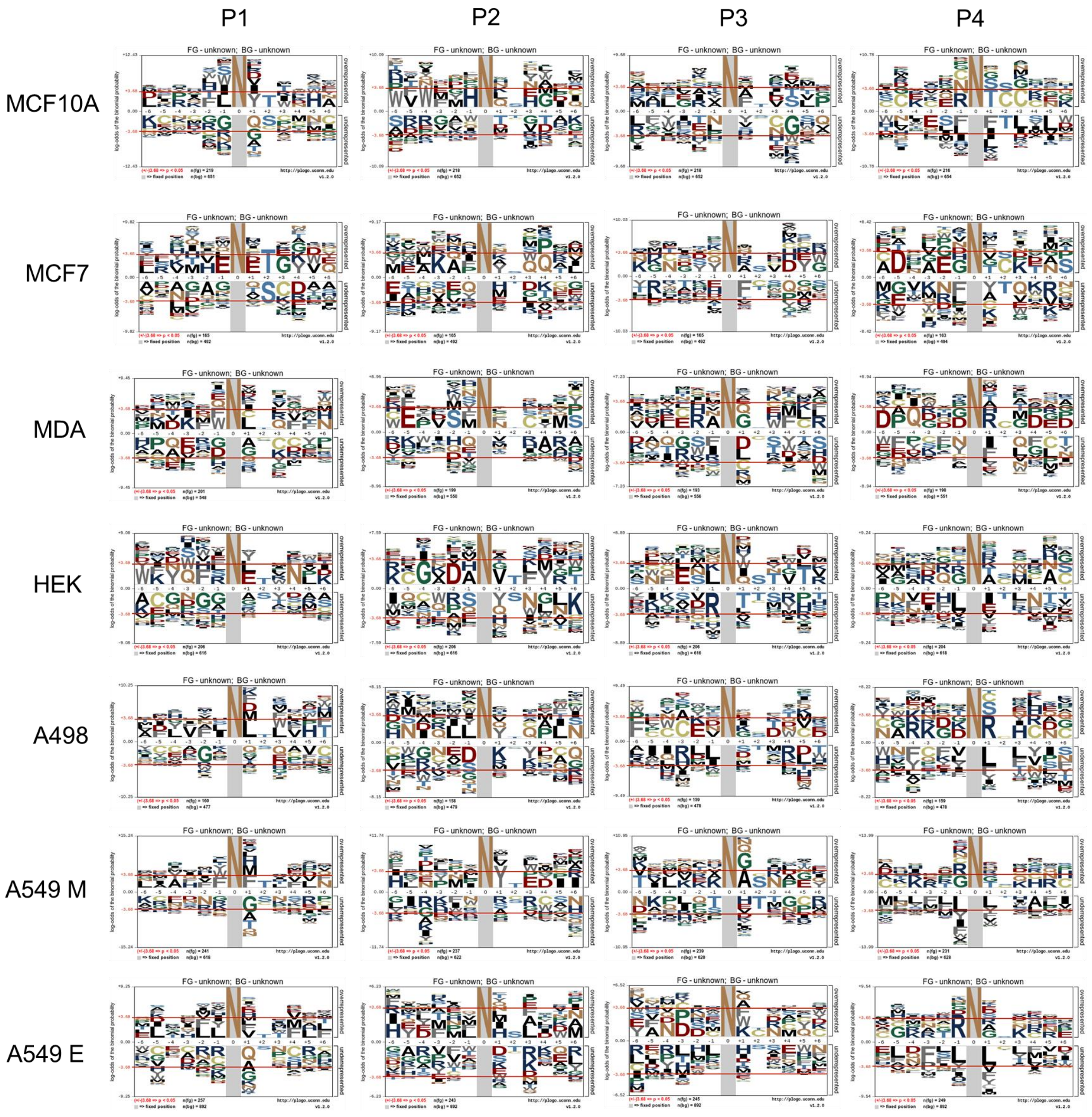

Fig. S8. Sequence motifs for core fucosylation sites with stoichiometry from low to high in various cell lines.

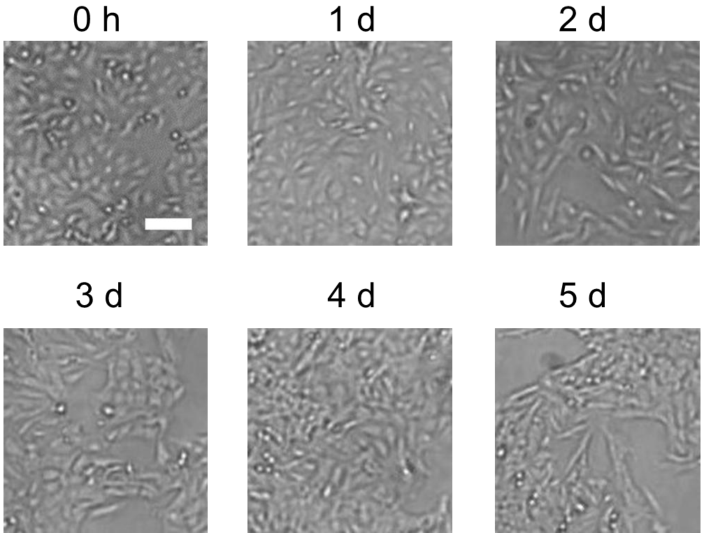

Fig. S9. A549 cell morphology change during EMT. Scale bar: 100 μm.

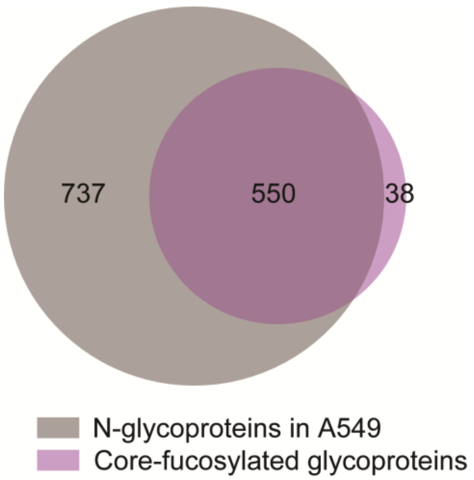

Fig. S10. Overlap of glycoproteins with core fucosylation with the whole N-glycoproteome in A549 cells.

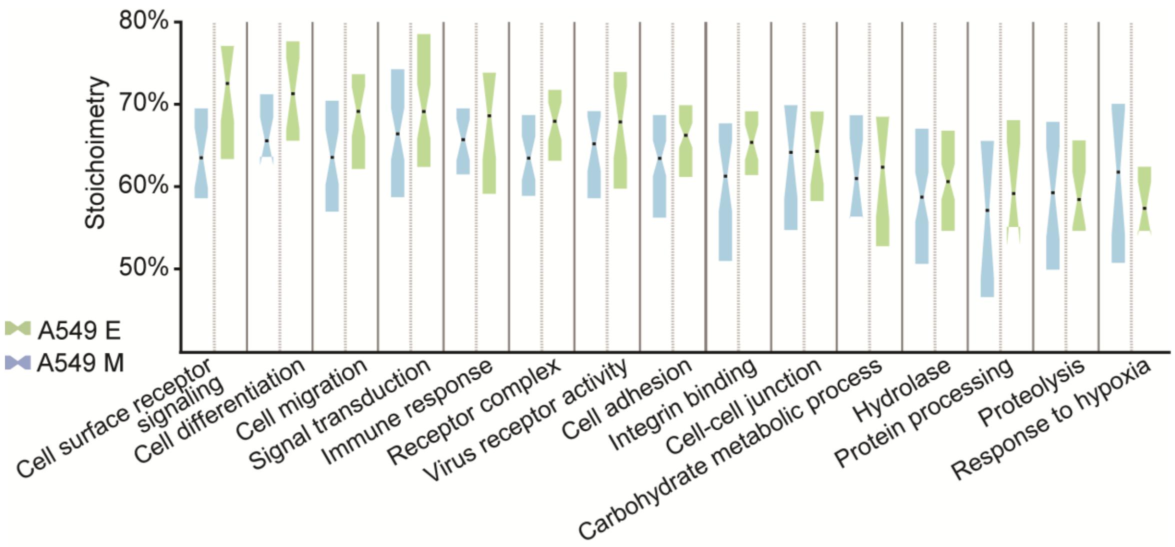

Fig. S11. Protein clustering results show the stoichiometry for core fucosylation sites on proteins associated with different GO terms. Box, 25th/75th percentiles; middle line, mean; the surrounding violin shape represents the density distribution of stoichiometry values.

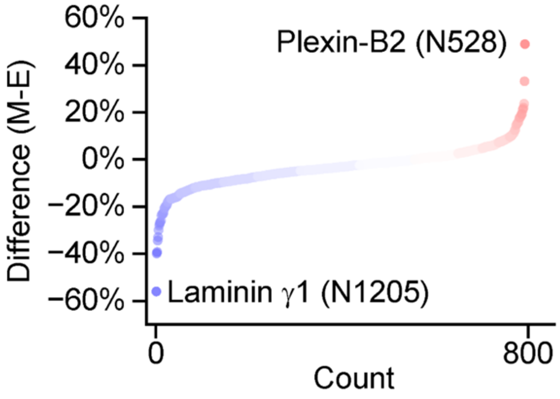

Fig. S12. Stoichiometry changes for the N-glycosylation sites with core fucosylation quantified in both the epithelial and mesenchymal states.
